# Acute stress disorder and the transition to posttraumatic stress disorder in children and adolescents: Prevalence, course, prognosis, diagnostic suitability, and risk markers

**DOI:** 10.1002/da.22602

**Published:** 2017-01-30

**Authors:** Richard Meiser‐Stedman, Anna McKinnon, Clare Dixon, Adrian Boyle, Patrick Smith, Tim Dalgleish

**Affiliations:** ^1^ Medical Research Council Cognition and Brain Sciences UnitCambridge; ^2^ Department of Clinical PsychologyUniversity of East AngliaNorwichUK; ^3^ Cambridge University Hospitals NHS Foundation Trust; ^4^ Institute of Psychiatry, Psychology and NeuroscienceKings College London; ^5^ Cambridgeshire and Peterborough NHS Foundation Trust

**Keywords:** anxiety/anxiety disorders, child/adolescent, life events/stress, PTSD/posttraumatic stress disorder, trauma

## Abstract

**Background:**

Early recovery from trauma exposure in youth is poorly understood. This prospective longitudinal study examined the early course of traumatic stress responses in recently trauma‐exposed youth, evaluated the revised DSM‐5 acute stress disorder (ASD) and PTSD diagnoses and alternative diagnoses, and identified risk factors for persistent traumatic stress.

**Method:**

Participants were 8‐ to 17‐year‐old emergency departments attendees exposed to single incident traumas. Structured clinical interviews were undertaken at 2 (*n* = 226) and 9 weeks (*n* = 208) posttrauma.

**Results:**

Using the revised criteria in DSM‐5, 14.2% met criteria for ASD at week 2 and 9.6% met criteria for PTSD at week 9. These prevalences were similar to the corresponding DSM‐IV diagnoses (18.6% ASD at week 2; 8.7% PTSD at week 9). Using the same diagnostic criteria (DSM‐IV or DSM‐5) across assessments (i.e., “2‐week PTSD”) suggested that caseness declined in prevalence by approximately half. Overlap between DSM‐IV and DSM‐5 ASD and DSM‐5 preschool child PTSD diagnoses was considerable. Two diagnoses were strongly predictive of corresponding week 9 diagnoses. Youth with ASD who subsequently had PTSD reported more negative alterations in cognition and mood at 2 weeks than those youth who did not develop PTSD.

**Conclusions:**

Youth exposed to single‐event traumas experience considerable natural recovery in the first months posttrauma. Using DSM‐5 criteria, ASD may not capture all clinically significant traumatic stress in the acute phase and is only moderately sensitive for later PTSD. Future research needs to address the role and etiology of negative alterations in cognition and mood symptoms.

## 1 INTRODUCTION

Trauma exposure in childhood and adolescence is common (Copeland, Keeler, Angold, & Costello, 2007), with a substantial minority of trauma‐exposed youth going on to develop PTSD (Alisic et al., 2014). PTSD may be chronic in youth, potentially lasting years (Morgan, Scourfield, Williams, Jasper, & Lewis, 2003; Yule et al., 2000). Given this potential for profound disruption of a young person's development, researchers have attempted to understand the etiology and evolution of chronic PTSD (Winston, Kassam‐Adams, & Garcia‐Espana, 2003), with a view to early intervention (Marsac, Donlon, & Berkowitz, 2014).

Elucidating the early course of PTSD reactions in youth is critical to these efforts. Several studies attest to considerable natural recovery in youth exposed to trauma (Meiser‐Stedman, Smith, Glucksman, Yule, & Dalgleish, 2008; Meiser‐Stedman, Yule, Smith, Glucksman, & Dalgleish, 2005), as with adults (Shalev & Freedman, 2005). Recent prospective longitudinal studies, based on questionnaire surveys of youth samples, have delineated recovery patterns among trauma‐exposed youth (Hong et al., 2014; Le Brocque, Hendrikz, & Kenardy, 2010). Although the majority experience no clinically significant traumatic stress, a “recovery” group experiences acute symptoms that improve without treatment, whereas a “chronic” group experiences persistent symptoms. Such studies suggest that much of this natural recovery is front‐ended, occurring within the first 2 to 3 months posttrauma (Le Brocque et al., 2010). Measuring the natural recovery that young populations undergo within the first weeks of a trauma would inform their clinical management, for example, when to commence treatment, and distinguishing acute responses that will resolve without intervention from reactions likely to be chronic.

An additional clinical issue concerns the optimal diagnostic tools to be used for assessing acute responses to trauma in children and adolescents. Acute stress disorder (ASD) was introduced into the DSM‐IV (American Psychiatric Association, 1994) to encapsulate acute traumatic stress reactions and identify individuals at high risk of developing later PTSD. For children and adolescents, DSM‐IV ASD has had some modest success in predicting cases who have PTSD at follow up (although negative predictive power and specificity are poor) (Kassam‐Adams & Winston, 2004), but the emphasis placed on the particular predictive validity of the dissociative symptom cluster was not supported (Meiser‐Stedman et al., 2005). Significant revisions to both the ASD and PTSD diagnoses were made for DSM‐5 (American Psychiatric Association, 2013) Both no longer require an intense subjective peritraumatic reaction (i.e., fear, helplessness, or horror). The DSM‐5 ASD diagnostic algorithm now ignores symptom clusters, instead simply stipulating that nine or more symptoms be present (from a single list delineated into intrusion, negative mood, dissociation, avoidance, and arousal categories). The DSM‐5 PTSD diagnosis retains its cluster‐based algorithm, but includes a new cluster covering negative alterations in cognition and mood (comprising many symptoms previously included within the avoidance cluster). No studies have investigated the prevalence and course of these significantly revised diagnostic algorithms in youth using structured interviews, nor examined how they function in comparison to the previous algorithms in capturing the evolution of the stress disorder response.

It has been suggested that ASD be seen as a way of identifying individuals who require immediate treatment, rather than as a tool for highlighting those at risk of developing later PTSD (Bryant, 2011). There is a need to examine the validity of DSM‐5 ASD within youth populations with respect to this putative revised function. Preliminary evaluations of the revised DSM‐5 ASD criteria suggest that the nine or more symptom requirement is too strict; three or four symptoms may be more appropriate (Kassam‐Adams et al., 2012). Although previous adult and youth studies suggest ASD should not be relied upon for assessing the likelihood of chronic traumatic stress, the clinical management of these groups would be greatly informed by understanding how predictive acute diagnoses are of later PTSD. The ability of ASD to predict broader mental health difficulties has also not been examined; this may present a further important function for ASD, given the frequent comorbidity of PTSD with other disorders (e.g., depression). Moreover, by exploring how groups of symptoms (i.e., reeexperiencing and avoidance) predict PTSD, it may be possible to identify clinical markers for chronicity as well as distinguishing normal and pathological stress reactions.

A nosological issue specific to older children and adolescents is that the PTSD diagnostic criteria are produced primarily with adult samples in mind. Epidemiological studies (Copeland et al., 2007) and treatment trials (Cohen, Deblinger, Mannarino, & Steer, 2004; Nixon, Sterk, & Pearce, 2012) for childhood PTSD have often used subsyndromal diagnostic criteria, rather than full DSM‐IV criteria, due to concerns that standard criteria may exclude cases with clinically significant traumatic stress. An age‐appropriate PTSD diagnosis for preschool children introduced in the DSM‐5 better reflects the reliance on parent reporting and the difficulties with observing some symptoms. Some data suggest that these amended criteria (predominantly reducing the DSM‐IV symptom requirement for avoidance from three to one) are also more appropriate for older children and adolescents than the adult criteria (Meiser‐Stedman et al., 2008; Scheeringa, Wright, Hunt, & Zeanah, 2006). It is important to evaluate which diagnostic algorithm—either the revised DSM‐5 PTSD or the new preschool child PTSD criteria—best captures the needs of these older trauma‐exposed youth.

A prospective longitudinal study of trauma‐exposed youth, focusing on the course of reactions over the first 2 months posttrauma, addressed these issues. Specifically, we sought to address the following questions:

1. What is the prevalence and course of traumatic stress (according to DSM‐IV and DSM‐5 algorithms) in the first months posttrauma?
2. How well does ASD predict PTSD?
3. How well do alternative acute diagnoses (i.e., “2‐week PTSD” and ASD with a lower symptom threshold) predict PTSD?
4. Are the DSM‐5 preschool child PTSD criteria also better for older children and adolescents?
5. How well do 2‐week diagnoses predict other psychopathology?
6. What are the acute symptom profiles of different recovery trajectories?

## 2 METHOD

### 2.1 Participants

Participants were consecutive child and adolescent attendees (8–17 years) at four emergency departments (EDs) in the East of England following single event trauma between September 3, 2010 and April 30, 2013. Attendances were considered traumas if they were consistent with the DSM‐5 PTSD definition of a trauma, that is, they involved the threat of death or serious injury. Traumas were considered single event if they were “one‐off” incidents unrelated to maltreatment or abuse. Exclusion criteria were inability to speak English, mental retardation, attendance resulting from deliberate self‐harm, under the care of social services or a child protection issue was related to the presentation, and moderate to severe traumatic brain injury (i.e., posttraumatic amnesia ≥24 hr). Youth were not offered any psychological or psychiatric intervention by the hospital EDs or the study team. By the 9‐week assessment, 18 families had had some contact with a professional (outside the hospital or study team) where their child's mental health was addressed to some extent. As these contacts were very brief (typically only one appointment), were often not traumatic stress‐focused, did not involve a mental health professional in many instances (e.g., a family doctor), were concerned mostly with pretrauma mental health concerns or trauma‐related physical injuries, and were *predictive* of PTSD at 9 weeks rather than being protective (*χ* ^2^ = 47.85, *P* < .0001), they were not considered further.

Research nurses identified 773 eligible children. Due to incomplete/inaccurate details, 168 (21.7%) could not be contacted. Of 605 families contacted, 315 (52.1%) declined to participate, 30 cases (5.0%) did not meet entry criteria, and 260 (43.0%) agreed to participate. Of these, 226 (37.4% of attendees contacted) completed the initial 2‐week assessment (days since trauma, *M* = 22.0, SD = 7.2); the remainder only completed the 9‐week assessment and are not included here.

There were no significant differences between participants (*n* = 260) and eligible nonparticipants (*n* = 483, including children who could not be contacted) with respect to age, sex, ethnicity, number of injuries, having a medical procedure in ED, being seen in resus, days admitted, previous attendances, head injury, or Glasgow Coma Scale scores (*P*s > .05). Relative to nonparticipants, participants were more likely to report greater pain, be admitted to hospital, be admitted to pediatric intensive care, have lost consciousness, have been assaulted, have received opiates, and have been intubated (all *P*s < .05).

Of the 226 participating children at 2 weeks (mean 14.1 years, SD = 2.9), 96 (42.5%) were female and 16 (7.1%) belonged to a minority ethnic group or were mixed race. Participants had experienced a motor vehicle collision (*n* = 104; 46.0%), an assault (*n* = 41; 18.1%), a dog attack (*n* = 10; 4.4%), serious accidental injuries (*n* = 70; 31.0%), and a medical emergency (*n* = 1; 0.4%). Forty‐eight (21.2%) sustained a fracture, 62 (27.4%) were admitted to hospital and 13 (5.8%) to an intensive care unit. Thirty‐nine (17.3%) received opiate mediation. Eighty‐six (38.1%) sustained a head injury during the trauma, whereas 26 (11.5%) lost consciousness during or shortly after the trauma. Nine (4.0%) were intubated at the scene of the trauma.

Two hundred and eight participants (92.0% of those who completed the two‐week assessment) completed a second assessment 9 weeks posttrauma (*M* = 67.5 days, SD = 11.7). There were no differences between youth who did or did not complete the week 9 assessment in terms of sex, age, or initial traumatic stress symptoms (*P*s > .15).

### 2.2 Measures

The Children's PTSD Inventory (CPTSDI) (Saigh et al., 2000) was used to assess ASD and PTSD. The CPTSDI is a youth‐report structured interview that possesses good internal consistency, interrater reliability, test–retest reliability, convergent validity, and discriminant validity (Saigh et al., 2000; Yasik et al., 2001). The CPTSDI was originally designed to assess for DSM‐IV PTSD; additional items were used to assess for dissociation symptoms (Meiser‐Stedman et al., 2005) and new symptoms relating to “negative alterations in cognition and mood” proposed for PTSD in DSM‐5 (available from first author). Thus, both DSM‐IV and DSM‐5 algorithms for ASD and PTSD were evaluated. Youth with clinically significant anxiety and depression were identified using self‐report questionnaire cut‐offs for the 38‐item Spence Child Anxiety Scale (scores ≥ 60; possible range 0–114) and the 13‐item Short Mood and Feelings Questionnaire (scores ≥ 8; possible range 0–26), respectively (Costello & Angold, 1988; Spence, 1998).

### 2.3 Procedure

The study was approved by the UK National Research Ethics Service, Cambridgeshire 1 Research Ethics Committee (10/H0304/11). The parents/caregivers of children meeting inclusion criteria were initially contacted by letter 2–4 days post‐ED attendance, then by telephone at 7–8 days to arrange the initial 2‐week assessment. Written, informed assent/consent from the child and their parent/carer was required for participation. Assessments were conducted via the telephone by graduate‐level psychologists. Diagnostic reliability was established via blind rating of interviews (23 [10%] of week 2 assessments, 21 [10%] of week 9 assessments) by two clinical psychologists with extensive experience of interviewing trauma‐exposed youth. For week 2 ASD, interrater reliability was excellent (κ = .83) for the DSM‐IV algorithm and perfect for the DSM‐5. There was 100% consensus on PTSD diagnosis status at week 9 (DSM‐IV and DSM‐5 algorithms). At 2 weeks, parents/carers answered additional questions about their child's ED attendance. Further information, for example, extent of injuries, was obtained from EDs. Follow‐up assessments were completed 9 weeks posttrauma.

### 2.4 Statistical analyses

Predictive abilities of different diagnostic thresholds were undertaken using positive/negative predictive values, sensitivity/specificity statistics, and logistic regression modeling; for these analyses, only the cases (*N* = 208) with full week 2 and 9 data were included. Where multiple between groups comparisons were made (i.e., when comparing recovery trajectories by different PTSD symptom clusters), adjustment for multiple comparison was made (reported in the text). Analyses were undertaken using SPSS (version 23).

## 3 RESULTS

### 3.1 What are the prevalence and course of traumatic stress in the first months posttrauma?

The prevalence of each DSM‐IV and DSM‐5 diagnosis, diagnostic subtype, and PTSD symptom cluster at 2 and 9 weeks are presented in Table 1. DSM‐5 ASD (week 2) and PTSD (week 9) were endorsed at rates (14.2% and 9.6%, respectively) similar to their DSM‐IV predecessors (18.6% and 8.7%). DSM‐5 PTSD‐defined symptom clusters were commonly endorsed at both week 2 (>37.6%) and week 9 (>22.1%). Impairment related to traumatic stress was reported more commonly than the proportion meeting any diagnostic threshold; indeed, it was reported at more than twice the rate of DSM‐5 ASD and PTSD. ASD with a reduced symptom threshold (four instead of nine symptoms, as suggested by previous research) (Kassam‐Adams et al., 2012) was met by over a quarter of participants.

**Table 1:** Prevalence of each symptom cluster and diagnosis by time point

	Time Point				
	Week 2 (*n* = 226)		Week 9 (*n* = 208)	
Diagnosis or Symptom Cluster	*n*	%	*n*	%	McNemar's Test
DSM‐5 PTSD symptom clusters^a^					
B. Reexperiencing	111	49.1	61	29.3	*P* < .0001
C. Avoidance	114	50.4	77	37.0	*P* < .0001
D. Cognitions and mood	95	42.0	46	22.1	*P* < .0001
E. Arousal	85	37.6	49	23.7	*P* < .0001
G. Impairment	70	31.0	40	19.3	*P* < .001
Dissociation	27	12.1	9	4.3	*P* < .0001
DSM‐5 diagnoses					
ASD	32	14.2	–	–	–
4+ symptoms	60	26.5	–	–	–
PTSD^b^	41	18.1	20	9.6	*P* < .002
Preschool PTSD^b^	45	19.9	20	9.6	*P* < .0001
DSM‐IV diagnoses					
Acute stress disorder	42	18.6	–	–	–
‘Two‐week PTSD/PTSD^b^	40	17.7	18	8.7	*P* < .001
Other psychopathology^c^					
Depression (M, SD)	5.32	5.83	4.48	5.53	*t*_194_ = 3.46, *P* < .001
Depression (above cut‐off)	52	25.0	48	23.4	*P* > .4
Anxiety (M, SD)	45.61	12.83	42.14	12.18	*t*_194_ = 5.66, *P* < .0001
Anxiety (above cut‐off)	39	18.8	25	12.2	*P* < .02

a For comparison over time, the PTSD symptom clusters are used here.

b “Two‐week PTSD” ignores the requirement that symptoms be present for at least 4 weeks.

c *N* = 208 at week 2, *N* = 205 at week 9; depression assessed using the Short Mood and Feelings Questionnaire, anxiety assessed using the Spence Child Anxiety Scale.

In order to make a valid comparison over time, “2‐week PTSD” diagnoses (i.e., ignoring the limitation that PTSD cannot be diagnosed within 4 weeks of a trauma) (Brewin, Andrews, & Rose, 2003; Meiser‐Stedman et al., 2005) were also derived for the 2‐week assessment. This showed that rates of youth meeting all PTSD symptom and impairment criteria roughly halved between the two assessments, regardless of whether DSM‐IV or DSM‐5 criteria were used; 2‐week PTSD/PTSD and each PTSD symptom cluster were significantly less frequently endorsed at week 9 than at week 2 (all *P*s < .03). The number of PTSD symptoms endorsed declined significantly over time (week 2: *M* = 5.32, SD = 5.39; week 9: *M* = 3.25, SD = 4.43; *t*[196] = 7.76, *P* < .0001, *d* = .59).

No diagnoses differed significantly by sex at either time point. No age group differences (i.e., 8–12 vs. 13–17 year olds) were observed at week 2, but at week 9, DSM‐IV and DSM‐5 PTSD were more likely to be endorsed by older participants (*χ* ^2^ > 4.66, *P*s < .04; see Supporting Information Tables S1 and S2).

### 3.2 How well does ASD predict PTSD?

The ability of ASD at week 2 to predict PTSD at week 9 is detailed in Table 2. Both DSM‐5 and DSM‐IV 2‐week ASD diagnoses were significantly predictive of respective later PTSD diagnoses, as indexed by regression statistics (odds ratios >24.21). Moreover, each yielded high negative predictive values (all >.97) and high specificity coefficients (>.87). Positive predictive values were modest (.48 for DSM‐5 and .37 for DSM‐IV). Importantly, 30% of DSM‐5 PTSD cases at week 9 had not met DSM‐5 ASD at week 2.

**Table 2:** Sensitivity, specificity, positive and negative predictive values, and regression statistics of week 2 diagnoses to predict week 9 traumatic stress diagnoses

		Regression Statistics							
Week 2 Predictor	Week 9 Outcome	χ^2^	*P*	Odds Ratio	Positive Predictive Value	Negative Predictive Value	Sensitivity	Specificity	Percentage Correctly Identified
DSM‐5									
ASD	PTSD	28.95	<.0001	26.91	0.48	0.97	0.70	0.92	89.9
4+ symptoms	PTSD	28.95	<.0001	15.79	0.30	0.97	0.80	0.80	79.8
“Two‐week PTSD”^a^	PTSD	36.55	<.0001	22.64	0.41	0.97	0.75	0.88	87.0
Preschool PTSD	Preschool PTSD	39.08	<.0001	26.08	0.39	0.98	0.80	0.87	86.1
DSM‐IV									
ASD (DSM‐IV)	PTSD (DSM‐IV)	34.58	<.0001	24.21	0.37	0.98	0.78	0.87	86.5
Either algorithm									
ASD	PTSD	35.59	<.0001	21.52	0.39	0.97	0.75	0.88	86.5

Note. Sensitivity = Likelihood that someone with a given diagnosis at week 9 would have met criteria for the relevant diagnosis at week 2. Specificity = Likelihood that someone without a given diagnosis at week 9 would also not have met criteria for the relevant diagnosis at week 2. Positive predictive value = Likelihood that someone with a given diagnosis at week 2 would have the relevant diagnosis at week 9. Negative predictive value = Likelihood that someone without a given diagnosis at week 2 would not have the relevant diagnosis at week 9.

a “Two‐week PTSD” this refers to an “early PTSD” algorithm, that is, ignores the requirement that symptoms be present for at least 4 weeks.

### 3.3 How well do alternative acute diagnoses predict PTSD?

The ability of two alternative acute diagnostic algorithms to predict 9‐week PTSD was examined: “2‐week PTSD” (Brewin et al., 2003) and ASD with a reduced symptom threshold (four symptoms instead of nine; Table 2) (Kassam‐Adams et al., 2012). The reduced symptom threshold ASD was a highly sensitive and specific predictor of 9‐week PTSD, but with a corresponding reduction in positive predictive power. “Two‐week PTSD” had similar predictive properties to ASD, but with a smaller positive predictive value and improved sensitivity.

### 3.4 Are the DSM‐5 preschool child PTSD criteria also better for older children and adolescents?

The prevalences of PTSD based on the DSM‐5 preschool criteria are presented in Table 1. Preschool PTSD was more commonly endorsed than other DSM‐5 diagnoses at 2 weeks, but by week 9 PTSD and preschool PTSD were endorsed at the same rate. Preschool PTSD, in predicting the same 9‐week diagnosis, yielded a positive predictive value comparable to ASD and PTSD, and good sensitivity and specificity. Comparison with DSM‐IV ASD and PTSD revealed no major differences in predictive ability.

Of 47 (20.8% of total sample) cases to meet threshold for any diagnosis (i.e., DSM‐5 or DSM‐IV ASD or preschool PTSD) at week 2, 32 met criteria for all three diagnoses, with a further 15 meeting just DSM‐IV ASD or preschool PTSD (see Supporting Information Fig. S1a for all overlap data). The agreement between DSM‐5 ASD and preschool PTSD was large (κ = .80), as was the agreement between DSM‐5 and DSM‐IV ASD (κ = .84).

At week 9, 20 participants (9.6%) met criteria for any diagnosis (i.e., DSM‐5 or DSM‐IV PTSD, preschool PTSD), of whom two cases met criteria for both DSM‐5 PTSD and preschool PTSD but not DSM‐IV PTSD (Supporting Information Fig. S1b). There was complete agreement between DSM‐5 PTSD and preschool PTSD (κ = 1.00), and very strong agreement between DSM‐5 and DSM‐IV PTSD (κ = .94).

### 3.5 How well do 2‐week diagnoses predict other psychopathology?

Descriptive data and numbers scoring above cut‐off for the self‐report measures of depression and anxiety are displayed in Table 1. Although scores on both measures improved significantly over time, the effect for anxiety was medium in size (Cohen's *d* = .41) while the effect size for depression was only small (Cohen's *d* = .25); this was mirrored in the small decrease in numbers reporting anxiety, and no significant change in numbers above cut‐off on our depression measure.

The abilities of 2‐week ASD diagnoses, 2‐week PTSD, and early preschool PTSD to predict 9‐week depression and anxiety (i.e., scoring above cut‐off) are presented in Table 3. Logistic regression suggested that each diagnosis was a significant predictor of depression and anxiety “caseness” (*P*s < .0001, odds ratios > 4.36). Positive predictive values were modest for depression (.47–.55) and weaker for anxiety (.32–.46). Negative predictive values were high for depression (.81–.85) and anxiety (.93–.95), as were specificity statistics (.88–.92 and .80–.92). Sensitivity statistics ranged slightly more widely (.31–.52 and .52–.68).

**Table 3:** Sensitivity, specificity, positive and negative predictive values, and regression statistics of week 2 diagnoses to predict week 9 depression and anxiety

	Regression Statistics							
Week 2 Predictor	χ^2^	*P*	Odds Ratio	Positive Predictive Value	Negative Predictive Value	Sensitivity	Specificity	Percentage Correctly Identified
Outcome: Depression at week 9								
ASD (DSM‐5)	14.18	<.0001	5.04	0.54	0.81	0.31	0.92	77.6
4+ symptoms	20.64	<.0001	5.01	0.47	0.85	0.52	0.82	75.1
ASD (DSM‐IV)	14.21	<.0001	4.36	0.49	0.82	0.38	0.88	76.1
“Two‐week PTSD” (DSM‐5)^a^	18.40	<.0001	5.40	0.53	0.83	0.40	0.89	77.6
Preschool PTSD	24.33	<.0001	6.53	0.55	0.84	0.46	0.89	78.5
Outcome: Anxiety at week 9								
ASD (DSM‐5)	25.60	<.0001	11.92	0.46	0.93	0.52	0.92	86.8
‐ 4+ symptoms	22.84	<.0001	8.50	0.32	0.95	0.68	0.80	78.5
ASD (DSM‐IV)	21.71	<.0001	8.69	0.38	0.93	0.56	0.87	83.4
“Early PTSD” (DSM‐5 PTSD)	22.54	<.0001	9.14	0.39	0.93	0.56	0.88	83.9
Preschool PTSD	20.84	<.0001	5.46	0.40	0.95	0.64	0.87	83.9

Note. Positive predictive value = Likelihood that someone with diagnosis at week 2 would have the relevant diagnosis at week 9. Negative predictive value = Likelihood that someone without a given diagnosis at week 2 would not go on to have the relevant diagnosis at week 9. Sensitivity = Likelihood that someone with diagnosis at week 9 would have previously met criteria for relevant diagnosis at week 2. Specificity = Likelihood that someone without diagnosis at week 9 would not have met criteria for the relevant diagnosis at week 2.

a “Two‐week PTSD” refers to an “early PTSD” algorithm, that is, ignores the requirement that symptoms be present for at least 4 weeks.

### 3.6 What are the acute symptom profiles of different recovery trajectories?

In order to identify whether particular week 2 symptom clusters were vulnerability factors for the development of PTSD, the week 2 symptom profiles of different stress response trajectories were explored using the DSM‐5 criteria (see Table 4). Three trajectories were considered: no 2‐week ASD, then no 9‐week PTSD (“resilient”), ASD then no PTSD (“recovery”), and ASD then PTSD (“persistent”) (Ginzburg et al., 2003). As the “delayed onset” trajectory (i.e., no ASD then PTSD) only comprised seven cases, this group was not considered further.

**Table 4:** Means and standard deviation for week 2 posttraumatic stress symptoms by trajectory

Symptom Cluster	Resilient (*n* = 173)	Recovered (*n* = 15)	Persistent (*n* = 14)	Delayed Onset (*n* = 6)	Total Sample (*n* = 208)
B. Reexperiencing (0–5)	0.73 (1.07)^a^	4.27 (0.70)^b^	4.36 (0.63)^b^	2.17 (1.60)	1.27 (1.63)
C. Avoidance (0–2)	0.59 (0.78)^a^	2.00 (0.00)^b^	1.93 (0.27)^b^	1.17 (0.98)	0.80 (0.87)
D. Cognition and mood (0–7)	1.16 (1.36)^a^	4.60 (1.30)^b^	6.00 (0.78)^c^	2.50 (1.87)	1.77 (1.97)
E. Arousal (0–6)	0.98 (1.16)^a^	4.07 (1.03)^b^	4.36 (1.01)^b^	2.83 (1.17)	1.48 (1.61)
Dissociation (0–2)	0.09 (0.34)^a^	0.47 (0.64)^b^	0.86 (0.95)^b^	0.50 (0.84)	0.18 (0.50)
Total symptoms (B + C + D + E)	3.46 (3.39)^a^	14.93 (1.91)^b^	16.64 (1.65)^b^	8.67 (4.37)	5.32 (5.39)

Possible range for each symptom cluster given in parentheses (first column). Standard deviations in parentheses (second to sixth columns). Different superscript characters represent significant post hoc differences, adjusted for multiple comparisons; delayed onset cases removed from post hoc analyses.

As data were skewed, nonparametric (Kruskal–Wallis *H*) tests were used for between groups comparisons; these indicated significant differences for each symptom cluster and total score (*P*s < .001) at 2 weeks. Post hoc tests (Mann–Whitney *U* with *P* values adjusted for multiple comparisons) revealed unsurprisingly that both the recovery and persistent groups reported more acute symptoms for each cluster than the resilient group (all *P*s < .0003). When comparing the recovery and persistent groups, a significant difference was only observed for acute negative alterations in cognitions and mood symptoms (*P* < .005; below adjusted threshold of .007); total symptom counts also showed no significant difference at this significance threshold. This finding was further supported by logistic regression modeling: two‐week cognitions and mood symptoms (Wald *χ* ^2^ = 4.41, *P* < .04), but not reexperiencing, avoidance, and arousal symptom clusters (Wald *χ* ^2^ < 2.35, *P*s > .13), accounted for unique variance in 9‐week PTSD.

## 4 DISCUSSION

This study was the first to track the early recovery (i.e., within 3 months posttrauma) of children and adolescents exposed to single‐event traumas using structured interviews, and to consider the validity of the DSM‐5 ASD and PTSD diagnoses. With respect to the course of traumatic stress responses, there was considerable improvement between assessments. Although the majority of participants did not reach diagnostic threshold at either assessment, experiencing some traumatic stress was common. The revised DSM‐5 ASD diagnosis was slightly more clinically conservative than its DSM‐IV incarnation, yielding fewer positive cases, but there were no clear differences in prevalence between DSM‐5 and DSM‐IV PTSD. With respect to predictive ability, youth who developed DSM‐5 ASD were at elevated risk of having DSM‐5 PTSD at week 9, but the sensitivity of acute diagnosis was not compelling. Shifting the diagnostic requirements (e.g., using “2‐week PTSD” or a reduced symptom count for ASD) led to modest improvements in sensitivity but at the cost of positive predictive ability.

The DSM‐5 preschool child PTSD diagnosis was more frequently endorsed than DSM‐5 ASD at week 2 but overlapped completely with DSM‐5 PTSD at week 9. Moreover, this diagnosis (at week 2) had similar predictive ability to DSM‐5 ASD and PTSD. Each 2‐week traumatic stress diagnosis significantly predicted marked anxious and depressive psychopathology at week 9, but was only modestly sensitive. The proportion of youth with clinically significant levels of depression did not show any recovery over time.

These findings speak to the early management of traumatic stress and associated mental health difficulties in youth, the nosology of PTSD in youth, and our theoretical understanding of PTSD in this age group. The present results provide clear confirmation of earlier preliminary questionnaire‐based research (Le Brocque et al., 2010). There is significant recovery from acute stress reactions within 2–3 months of the trauma, even without formal intervention. Whether very early intervention (i.e., within 2 months of a trauma) is warranted or even appropriate needs careful consideration. Future early intervention research should address forms of intervention likely to outperform (and not disrupt) such early recovery.

ASD may function as a tool for highlighting youth at very elevated risk of developing PTSD for whom treatment may be indicated (with nearly a half of cases going to develop PTSD). However, it is important to note that a significant proportion of youth who develop PTSD will not have met criteria for the acute disorder. As with its DSM‐IV conceptualization, DSM‐5 ASD cannot be relied upon as a way of identifying children and adolescents will develop PTSD. Moreover, given the discrepancy between the prevalence of ASD—based on both the DSM‐IV and particularly the DSM‐5 algorithms—and the number of participants reporting impairment, the acute diagnosis has limited utility for identifying clinically significant traumatic stress in the acute phase in youth. This insensitivity to acute impairment is at odds with one of the main purposes for ASD, that is, identifying cases that warrant treatment in the acute phase (Bryant, Friedman, & Spiegel, 2011). This weakness largely stems from the large number of symptoms required; reducing the threshold to four symptoms (while retaining the impairment criterion) doubled the prevalence of identified disorder. Although DSM‐5 ASD may be a stronger indicator for a transition from an acute reaction to persistent PTSD, early PTSD and a reduced symptom version of ASD have the advantage of being more sensitive to both acute impairment and later PTSD. “Two‐week” PTSD would also be a more parsimonious assessment option, being reliant on the more widely used assessment tools for PTSD.

Despite the changes to PTSD in DSM‐5, there was little evidence of substantial changes in prevalence relative to DSM‐IV. However, this study demonstrated the importance and utility of the new negative alterations in cognitions and mood PTSD symptom cluster, that is, its ability to delineate different symptom trajectories and to uniquely predict later PTSD.

Consistent with earlier studies that have addressed its utility in older children and adolescents, the DSM‐5 preschool PTSD diagnosis had similar predictive validity and completely overlapped with DSM‐5 PTSD at the week 9 assessment. This supports the continued use of the simplified (preschool) diagnostic criteria for all trauma‐exposed youth in treatment trials (Cohen et al., 2004; Nixon et al., 2012), and raises the issue of whether the DSM‐5 preschool child criteria should be routinely used with all youth. Again, the decision about whether to focus on such a tool would be informed by the function and context of an assessment and would involve balancing different demands. Using the preschool child PTSD criteria would make for a simpler assessment, reducing the burden on young people and assessors, which would be of particular important when working with younger children.

The evaluation of the ability of the ASD diagnosis to predict other nontraumatic stress psychopathology warrants further discussion. This study has demonstrated that, while acute traumatic stress reactions are one pathway to PTSD and other significant mental health difficulties, it is not the only acute presentation that warrants consideration. Clinically significant levels of depression in particular were present in a significant minority, demonstrated comparatively little natural recovery and were not predicted by DSM‐IV or DSM‐5 ASD in over 60% of cases. ASD does not function as a “catch‐all” for posttraumatic mental health difficulties, and screening for depression—even if some depression may have preexisted the trauma—is warranted.

Theoretically, these data support viewing PTSD in youth as lying on a spectrum with the early traumatic stress reactions experienced by many children and adolescents exposed to a single‐event trauma (indeed, some PTSD symptoms in the first weeks posttrauma was the modal response), with natural recovery (equivalent to a medium effect size in this study) in the initial months posttrauma. The presence of high numbers of symptoms now encapsulated within the negative alterations in cognitions and mood symptom cluster of PTSD distinguishes an acute response more likely to become chronic from a “normal” nonclinical response. More research is needed to explore why this symptom cluster may become so prevalent in some youth.

Clinically, these data are important for several reasons, aside from the assessment issues outlined above. First they help to normalize responses to trauma, since there is not a clear‐cut distinction between “normal” and pathological responses to trauma in youth. Second, these findings speak to the issue of when to initiate intervention. Families and clinicians may consider these results in the acute phase, and explore whether a watchful waiting approach or active treatment may be more appropriate, according to their own circumstances and preferences. Third, these findings may help to clarify the clinical question for practitioners and the young people they work with; rather than focusing on why has a young person developed PTSD, or what is maintaining this condition, it may be beneficial to think in terms of what is preventing the recovery seen in many other youth. Fourth, these data are an important reminder of the need to consider posttraumatic mental health beyond PTSD.

This study has several limitations. The study was restricted to youth exposed to discrete traumas that had limited wider impact; further research is needed in youth affected by large‐scale disasters, with longer term follow up and closer attention to depression. Self‐report questionnaire measures were used for assessing anxiety and depression, rather than interviews. The focus of the study was children's and adolescents’ early reactions to trauma, but more research is required that addresses the long‐term sequelae of early responses to trauma.

## Supporting information

Supporting Information

Supporting Information

Supporting Information

Supporting Information

Supporting Information

Supporting Information

Supplementary FIGURE S1. Diagnostic overlap at each assessment. (a) Acute (week two) assessment. (b) Week nine follow up assessment

Supplementary TABLE S1 Prevalence of diagnoses and criteria, by sex and age group at week two
Supplementary TABLE S2 Frequency of diagnoses and criteria, by sex and age group at week nine
