# Supplementary material for: Acute stress disorder and the transition to posttraumatic stress disorder in children and adolescents: Prevalence, course, prognosis, diagnostic suitability, and risk markers
Source: Depress Anxiety. 2017 Jan 30;34(4):348–55. doi: 10.1002/da.22602 (PMC5381710; doi:10.1002/da.22602)
Supplement: Supplementary file 7 — Supplementary FIGURE S1. Diagnostic overlap at each assessment. (a) Acute (week two) assessment. (b) Week nine follow up assessment [file DA-34-348-s007.docx]

Supplementary Figure 1. Diagnostic overlap at each assessment.

Supplementary Figure 1a. Acute (week two) assessment


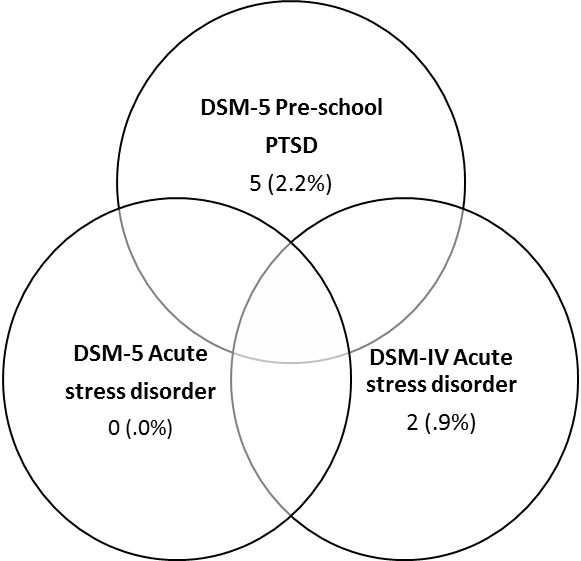


8 (3.5%)

**No diagnosis**

179 (79.2%)

32 (14.2%)

0 (.0%)

0 (.0%)

Supplementary Figure 1b. Week nine follow up assessment


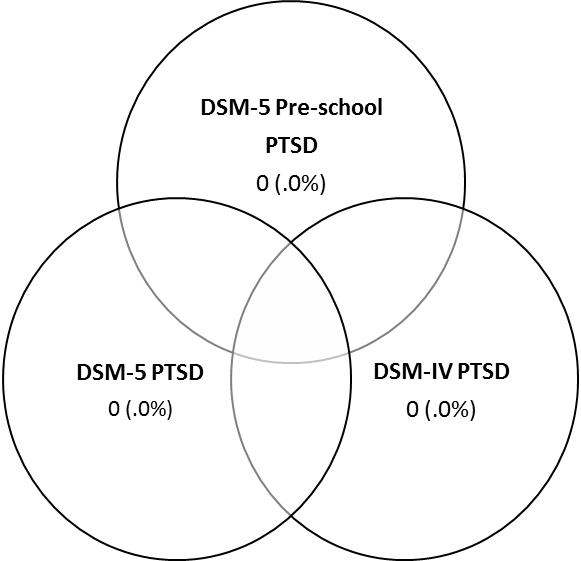


18 (8.7.%)

**No diagnosis**

188 (90.4%%)

2 (.9%)

0 (.0%)

0 (.0%)
