# Supplementary material for: Acute stress disorder and the transition to posttraumatic stress disorder in children and adolescents: Prevalence, course, prognosis, diagnostic suitability, and risk markers
Source: Depress Anxiety. 2017 Jan 30;34(4):348–55. doi: 10.1002/da.22602 (PMC5381710; doi:10.1002/da.22602)
Supplement: Supplementary file 8 — Supplementary TABLE S1 Prevalence of diagnoses and criteria, by sex and age group at week two Supplementary TABLE S2 Frequency of diagnoses and criteria, by sex and age group at week nine [file DA-34-348-s008.docx]

*Supplementary Table 1. Prevalence of diagnoses and criteria, by sex and age group at week two.*

|  | Sex | | | | Age group | | | |  | |
| --- | --- | --- | --- | --- | --- | --- | --- | --- | --- | --- |
|  | Male  (n=130) | | Female  (n=96) | | 8-12 years  (n=80) | | 13-17 years  (n=146) | | All  (n=226) | |
|  | n | % | n | % | n | % | n | % | n | % |
| *DSM-5 PTSD symptom clusters ^a^* | | | | | | | | | | |
| B. Re-experiencing | 57 | 43.8^†^ | 54 | 56.3^†^ | 40 | 50.0 | 71 | 48.6 | 111 | 49.1 |
| C. Avoidance | 63 | 48.5 | 51 | 53.1 | 51 | 63.8^†^ | 63 | 43.2^†^ | 114 | 50.4 |
| D. Cognitions and mood | 52 | 40.0 | 43 | 44.8 | 30 | 37.5 | 65 | 44.5 | 95 | 42.0 |
| E. Arousal | 48 | 36.9 | 37 | 38.5 | 29 | 36.3 | 56 | 38.4 | 85 | 37.6 |
| G. Impairment | 36 | 27.7 | 34 | 35.4 | 24 | 30.0 | 46 | 31.5 | 70 | 31.0 |
| Dissociation | 16 | 12.5 | 11 | 11.5 | 7 | 8.9 | 20 | 13.8 | 27 | 12.1 |
| *DSM-5 diagnoses* |  |  |  |  |  |  |  |  |  |  |
| ASD | 17 | 13.1 | 15 | 15.6 | 9 | 11.3 | 23 | 15.8 | 32 | 14.2 |
| - 4+ symptoms | 32 | 24.6 | 28 | 29.2 | 21 | 26.3 | 39 | 26.7 | 60 | 26.5 |
| ‘Early PTSD’ | 22 | 16.9 | 19 | 19.8 | 13 | 16.3 | 28 | 19.2 | 41 | 18.1 |
| Preschool PTSD | 24 | 18.6 | 21 | 21.9 | 13 | 16.3 | 32 | 21.9 | 45 | 19.9 |
| *DSM-IV diagnoses* |  |  |  |  |  |  |  |  |  |  |
| ASD | 22 | 16.9 | 20 | 20.8 | 12 | 15.0 | 30 | 20.5 | 42 | 18.6 |
| ‘Early PTSD’ | 23 | 17.7 | 17 | 17.7 | 12 | 15.0 | 28 | 19.2 | 40 | 17.7 |

Note. ^†^ Indicates significant age- or sex-related difference within the same row.

*Supplementary Table 2. Frequency of diagnoses and criteria, by sex and age group at week nine.*

|  | Sex (n, %) | | | | Age group (n, %) | | | |  | |
| --- | --- | --- | --- | --- | --- | --- | --- | --- | --- | --- |
|  | Male  (n=119) | | Female  (n=89) | | 8-12 years  (n=72) | | 13-17 years  (n=136) | | All (n, %)  (n=208) | |
| *DSM-5 PTSD symptom clusters ^a^* | | | | | | | | | | |
| B. Re-experiencing | 28 | 23.5^†^ | 33.0 | 37.1^†^ | 20 | 27.8 | 41 | 30.1 | 61 | 29.3 |
| C. Avoidance | 42 | 35.3 | 35.0 | 39.3 | 35 | 48.6^†^ | 42 | 30.9^†^ | 77 | 37.0 |
| D. Cognitions and mood | 26 | 21.8 | 20.0 | 22.5 | 8 | 11.1^†^ | 38 | 27.9^†^ | 46 | 22.1 |
| E. Arousal | 26 | 22.0 | 23.0 | 25.8 | 10 | 13.9^†^ | 39 | 28.9^†^ | 49 | 23.7 |
| G. Impairment | 25 | 21.2 | 15.0 | 16.9 | 9 | 12.5 | 31 | 23.0 | 40 | 19.3 |
| Dissociation | 6 | 5.1 | 3.0 | 3.4 | 2 | 2.8 | 7 | 5.2 | 9 | 4.3 |
| *DSM-5 diagnoses* |  |  |  |  |  |  |  |  |  |  |
| PTSD | 12 | 10.1 | 8.0 | 9.0 | 2 | 2.8^†^ | 18 | 13.2^†^ | 20 | 9.6 |
| Preschool PTSD | 12 | 10.1 | 8.0 | 9.0 | 2 | 2.8^†^ | 18 | 13.2^†^ | 20 | 9.6 |
| *DSM-IV diagnoses* |  |  |  |  |  |  |  |  |  |  |
| PTSD | 11 | 9.2 | 7.0 | 7.9 | 2 | 2.8^†^ | 16 | 11.8^†^ | 18 | 8.7 |

Note. ^†^ Indicates significant age- or sex-related difference within the same row.
